# Ventrogluteal site as the preferred choice for pediatric intramuscular injections: impact on pain and fear

**DOI:** 10.1590/1806-9282.20241782

**Published:** 2025-05-02

**Authors:** Hongmei Xuan, Lifeng Meng, Peiyu Chen

**Affiliations:** 1 Zhuji People's Hospital, Department of Pain – Zhuji, China.; 2 Zhuji People's Hospital, Department of Renal Rheumatology – Zhuji, China.; 3 Zhuji Hospital of Traditional Chinese Medicine, Department of Anesthesiology – Zhuji, China.

Dear Editor,

I read with great interest the study by Tiryaki et al., examining the effects of different intramuscular injection (IMI) sites—specifically, the ventrogluteal (VG) and vastus lateralis (VL) sites—on pain and anxiety in young children^1^. This research offers valuable insights into methods for reducing discomfort associated with IMIs, a routine yet often distressing experience for pediatric patients. The selection of injection site in clinical practice has long been a topic of considerable interest, especially in pediatrics, where children's reactions to pain and anxiety can complicate standard medical procedures and influence their overall healthcare experience.

The authors provide compelling evidence that the VG site markedly reduces pain, fear, and crying duration in children compared to the VL site. The study employs a robust methodology, utilizing a randomized controlled trial (RCT), to compare the two sites across metrics, such as pain—measured by the Wong-Baker Faces Pain Rating Scale—and fear, assessed through the Children's Fear Scale (CFS). This rigorous approach lends credibility to the study's conclusions, making a strong case for favoring the VG site as the preferred choice for pediatric IMIs.

The effectiveness of the VG site in reducing pain and distress likely stems from its distinct anatomical and physiological features^2^. Unlike other areas, the VG site is free of major blood vessels and nerves, lowering the risk of accidental injury. Additionally, its thinner subcutaneous layer allows for more efficient absorption of medications, reducing prolonged irritation. These characteristics jointly contribute to less immediate pain and a shorter duration of post-injection discomfort. In contrast, the VL site is situated near denser muscle tissues and bones, potentially contributing to higher pain scores and longer crying times in this group. Children receiving injections at the VL site showed a mean pain score of 7.60 compared to 5.49 in the VG group, a statistically significant difference that underscores the VG site's advantages for pain management.

The study's findings align with the existing literature on the benefits of the VG site for IMIs in adults, where it has been linked to lower pain levels and fewer complications compared to sites like the dorsogluteal (DG) region^3^. However, research specifically focused on pediatric populations remains limited, particularly studies addressing subjective outcomes such as fear and crying duration—key indicators of a child's comfort during procedures. This study therefore fills an important gap in pediatric nursing by providing empirical support for the VG site as a safer and more comfortable choice for children.

Interestingly, the study also examines the psychological aspect of IMIs by exploring fear, a common response in children facing medical procedures. Anticipatory anxiety, often heightened when children can see the needle approaching, exacerbates this fear^4^. The VG site, positioned on the lateral side of the hip, is typically outside the child's direct line of sight during the injection, potentially contributing to lower fear scores. Children in the VG group indeed reported significantly less fear both during and after the procedure compared to those in the VL group. By minimizing visible exposure to the needle, this site likely helps alleviate distress and reduce anticipatory anxiety, highlighting the VG site's advantages from both physical and psychological perspectives.

The findings of the study are not only meaningful but also offer practical benefits for clinical practice. Managing fear and anxiety in children during routine procedures is a common challenge in pediatric nursing. Utilizing the VG site could potentially reduce the time needed for IMIs by decreasing crying and recovery duration, thereby streamlining workflow in high-demand settings like emergency departments. Moreover, using the VG site can create a more positive experience for young patients, who are especially sensitive to pain and fear in medical contexts. Reducing distressing experiences in early childhood may positively shape a child's long-term perception of health care, helping alleviate anxiety about future medical visits.

However, as the authors note, there are limitations to this study. The sample size was restricted to 83 participants from a single center, limiting the generalizability of the findings until further validation in larger and more diverse pediatric populations. Additionally, the study focused on children aged 4–6 years, highlighting the need for further research to determine whether the benefits of the VG site extend to other pediatric age groups, such as infants or older children. Since muscle mass and fat distribution vary with age, examining whether the VG site remains optimal across developmental stages would be valuable. Furthermore, replicating this study in diverse clinical settings—including outpatient clinics and primary care facilities—could help establish the VG site's practicality and effectiveness beyond emergency care environments.

Future research could also explore the impact of parental involvement and distraction techniques in reducing procedural anxiety. Involving parents in comforting their child or incorporating distraction tools—such as toys or visual entertainment—may further alleviate pain and fear, complementing the choice of the injection site. Combining optimal site selection with these supportive strategies could create a more comprehensive approach to pain management in pediatric nursing, particularly for procedures like IMIs, which are often distressing for young patients.

In conclusion, Tiryaki et al. provide valuable insights into pain management for pediatric IMIs. By showing that the VG site is associated with reduced pain, fear, and crying duration, they present a compelling case for adopting the VG site as standard practice in pediatric IMIs. Their findings endorse the VG site as the preferred option, promoting atraumatic care practices in pediatric nursing and potentially influencing clinical protocols for IMIs in children. To expand on these findings, I encourage future research to examine the VG site's effectiveness across various pediatric age groups and in diverse healthcare settings. Such studies could further confirm the VG site's role in enhancing procedural comfort and fostering a positive healthcare experience for young patients.
